# Poster Session I - Poster of Distinction I - A19 MICROBIOTA-MEDIATED IMPACT OF CHRONIC STRESS ON INTESTINAL CRYPT STEM-CELL CYCLING AND PANETH-CELL PROLIFERATION

**DOI:** 10.1093/jcag/gwaf042.019

**Published:** 2026-02-13

**Authors:** X Bai, E Ihara

**Affiliations:** Medicine, McMaster University Faculty of Health Sciences, Hamilton, ON, Canada; Kyushu Daigaku, Fukuoka, Fukuoka Prefecture, Japan

## Abstract

**Background:**

Gut microbiota are key regulators of epithelial renewal and barrier defense. Chronic stress perturbs the gut–brain–microbiota axis (HPA, vagal, immune pathways), alters microbial communities, and can compromise epithelial integrity.

**Aims:**

To determine how chronic stress–induced dysbiosis affects the small-intestinal crypt stem-cell niche and Paneth-cell function.

**Methods:**

Six–eight-week-old mice underwent daily restraint stress (1 h/day, 7 days). Anxiety-like behavior was assessed with the dark/light transition test. Paracellular permeability was quantified in Ussing chambers. Middle-jejunum mucosa/submucosa (after muscle removal) was processed for RNA-seq; immunofluorescence probed OLFM4 (stem cells) and proliferation markers Ki-67 and p-H3. To model glucocorticoid signaling, dexamethasone (synthetic steroid) or metyrapone (11β-hydroxylation inhibitor) was administered. To test microbiota involvement, broad-spectrum antibiotics were given before and throughout stress exposure. Fecal communities were profiled by 16S rRNA analysis.

**Results:**

Chronic restraint stress produced anxiety-like behavior and dysbiosis, with increased *Streptococcus* and decreased *Bifidobacterium*. Barrier dysfunction was evident as increased small-intestinal epithelial permeability, accompanied by reduced villus and crypt length in the jejunum. RNA-seq demonstrated downregulation of Paneth-cell and stem-cell markers in jejunal mucosa. Immunofluorescence confirmed reduced OLFM4, Ki-67, and p-H3 within crypts. Ablation of gut microbiota with antibiotics attenuated the stress-induced downregulation of stem-cell markers but did not alter the Paneth-cell marker changes. Dexamethasone mimicked the effects of restraint stress on crypt stem cells and Paneth cells, whereas metyrapone attenuated both.

**Conclusions:**

Chronic stress drives microbiota changes that accompany anxiety-like behavior and barrier dysfunction. Epithelial villus/crypt alterations occur in a microbiota-dependent manner, with glucocorticoid signaling contributing to these effects. These findings delineate a microbiota-linked pathway by which stress impairs epithelial renewal and differentiation, with implications for stress-related intestinal disorders.

**Funding Agencies:**

McMaster Farcombe institute start-up grant for new faculty

